# Carcinoma developing in a fibroadenoma in a woman with a family history of breast cancer: a case report and review of literature

**DOI:** 10.1186/1757-1626-2-9348

**Published:** 2009-12-18

**Authors:** Chintamani, Rohan Khandelwal, Megha Tandon, K Yashwant, Pranjal Kulshreshtha, Tushar Aeron, Dinesh Bhatnagar, Anju Bansal, Sunita Saxena

**Affiliations:** 1 Department of Surgery, Vardhman Mahavir Medical College, Safdarjang Hospital, New Delhi-110023, India; 2 Institute of Pathology, Indian Council Of Medical Research, Safdarjang Hospital Campus, New Delhi, India

## Abstract

**Introduction:**

Malignant transformation of a fibroadenoma is rare with only about 100 cases reported in the world literature. Fibroadenoma occurring in middle aged woman with a strong family history of breast or ovarian cancer should be investigated with a high suspicion for malignancy.

**Case presentation:**

A 35-year- old Indian lady operated previously for fibroadenoma of the right breast presented with a recurrent lump at the same site. She had a strong family history of breast carcinoma. Mammography and trucut biopsy was suggestive of infiltrating duct carcinoma. She was managed by lumpectomy and axillary lymph node dissection with a satisfactory outcome. There was no evidence of BRCA-1, BRCA-2 mutation on immunohistochemistry.

**Conclusion:**

Malignant change in a pre-existing fibroadenoma is rare, however in a middle aged woman with a family history of breast cancer it should be suspected. In the absence of any definite clinical and radiological criteria of diagnosing malignant change in a fibroadenoma, high suspicion index is mandatory. The management and outcome depends on the stage and grade of presentation.

## Introduction

A carcinoma arising in a pre-existing fibroadenoma is a rare occurrence with about 100 cases reported in the world literature [1,2]. The diagnosis is usually a histological surprise [3-5]. Although malignant transformation in a fibroadenoma is rare, high suspicion index in middle aged women with fibroadenoma and associated risk factors like strong family history and/or BRCA-1, BRCA-2 mutation is recommended [6-8]. Since there are no definite clinical or radiological criteria of diagnosing carcinoma developing in a fibroadenoma, histopathological examination of all fibroadenomas should be performed routinely to rule out malignancy.

## Case presentation

**Figure 1:**
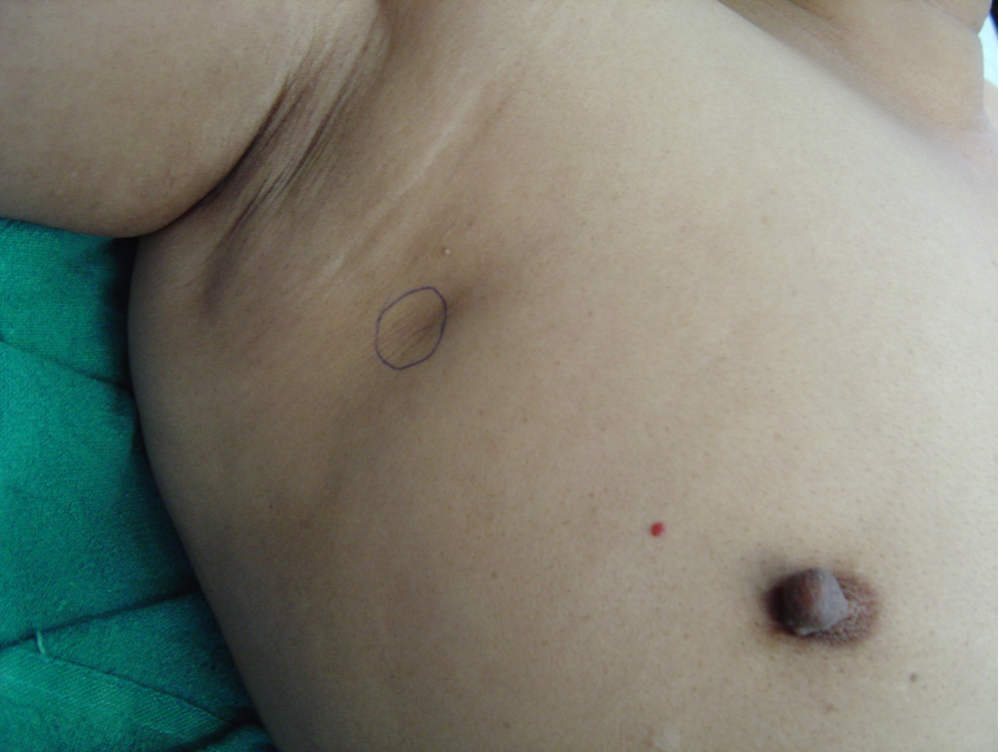
**The clinical picture showing the location of the lump and the previous operative scar**.

A -35- year old Indian lady presented with a recurrent lump in her right breast of two months duration. She had undergone surgery for the lump at the same site at a University college hospital nine months previously with histopathological diagnosis of fibroadenoma. Examination revealed a non-tender, mobile, hard lump measuring 2 × 3 cm in the upper and outer quadrant of the right breast (Figure 1). There was a 1 × 2 cm mobile axillary lymph node palpable on the right side.

**Figure 2:**
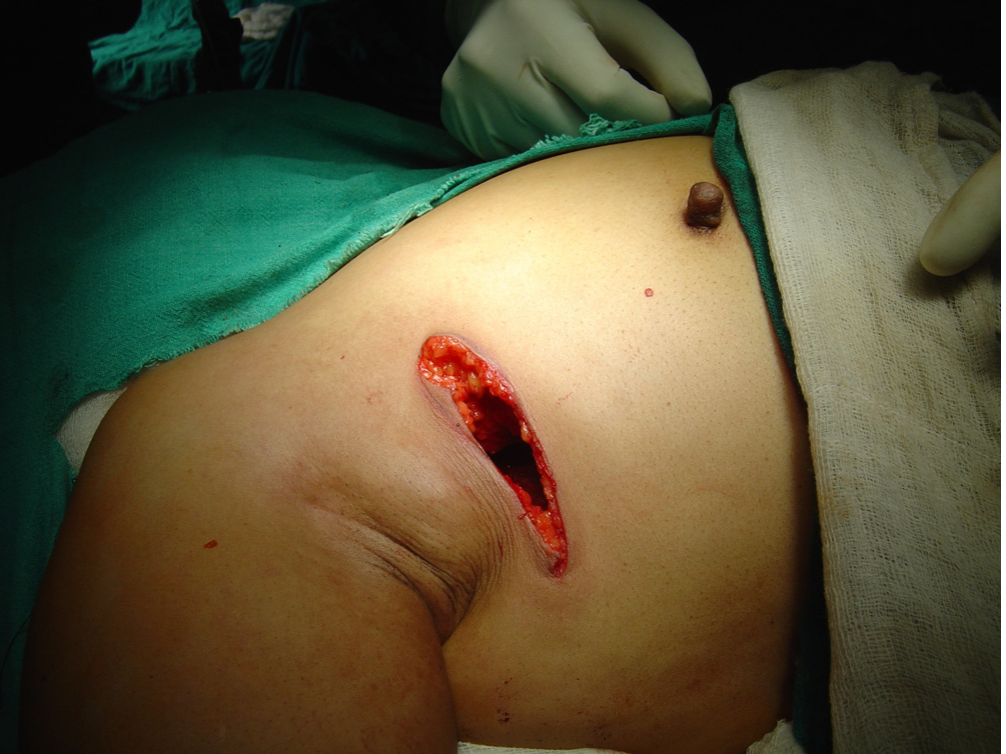
**The lumpectomy and axillary dissection being done through one incision**.

**Figure 3:**
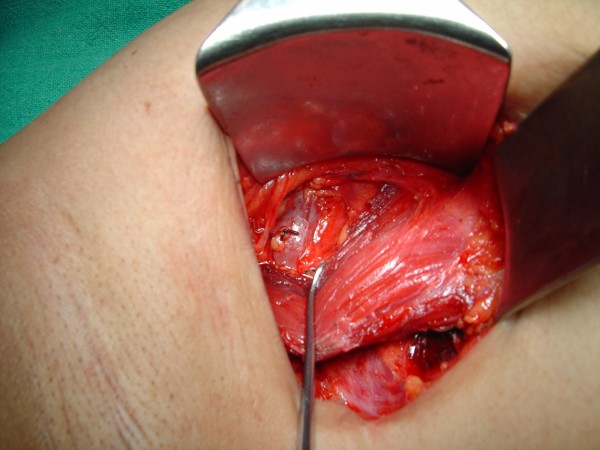
**The retracted pectoralis minor showing the axillary clearance of the level-III group of axillary lymph nodes**.

**Figure 4:**
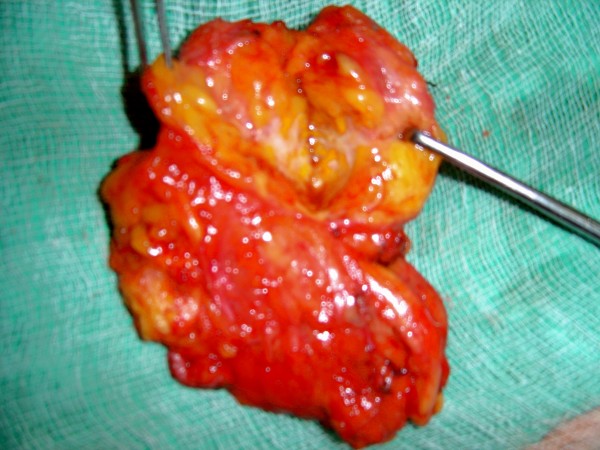
**The resected specimen**.

There was a significant family history (first degree relative of the patient had undergone surgery for infiltrating duct carcinoma of the breast one year previously). The mammogram was suggestive of carcinomatous changes in the lump and the trucut biopsy confirmed the diagnosis of malignancy (infiltrating duct carcinoma). She was treated by BCT (breast conserving therapy/surgery) in the form of lumpectomy and axillary lymph node dissection including levels-I, II, III (Figures 2, 3, 4).

**Figure 5:**
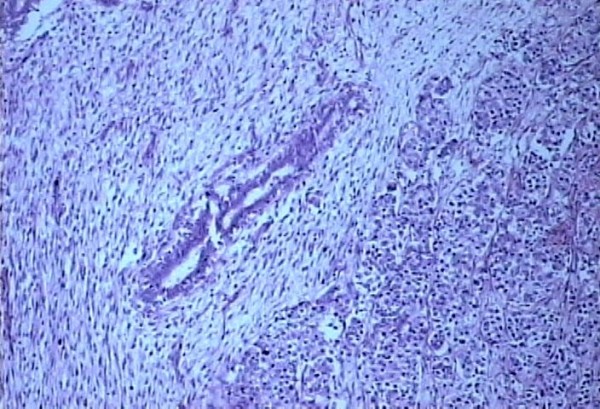
**Photomicrograph showing features of fibroadenoma and infiltrating duct carcinoma coexisting in one histological section (10×)**.

**Figure 6:**
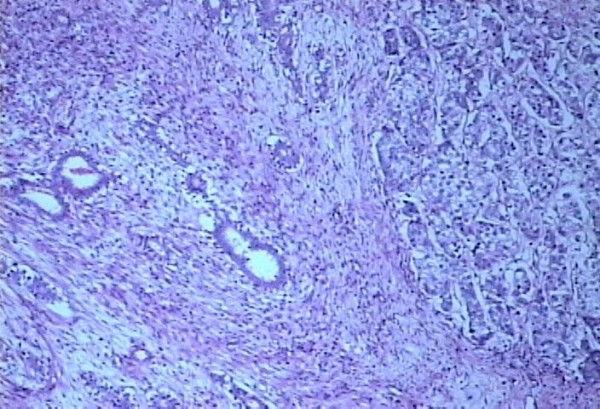
**Photomicrograph showing fibroadenoma (pericanalicular pattern) along with infiltrating duct carcinoma NOS Grade III**. In the same section of the tumor (40×).

The histopathological examination of the specimen revealed R0 resection (microscopic freedom from the disease). It showed Fibroadenoma (pericanalicular pattern) along with infiltrating ductal carcinoma NOS Grade III coexisting in the same histological section (Figures 5, 6). Two lymph nodes were positive for metastatic deposits. ER, PR receptors were positive and HER-2 neu receptor, p-glycoprotein status was negative on immunohistochemistry and there was no evidence of BRCA-1 or BRCA-2 mutation.

She was subjected to adjuvant chemotherapy (Cyclophosphamide, Adriamycin, 5-fluouracil regime), radiotherapy and hormone therapy in standard doses. Patient had an uneventful recovery and follow up of three years is satisfactory.

## Discussion

Carcinoma developing in a fibroadenoma is rare with only over hundred cases reported in the world literature. The reported mean age in various case series is 42.5 years, which is about 20 years later than the peak age of occurrence of fibroadenoma [1]. Therefore one needs to have a high suspicion index for malignancy in a case of fibroadenoma in an older woman particularly in one with associated risk factors like strong family history as in the reported case.

Although the fibroadenomas are not usually considered a risk factor for carcinoma but there are views to the contrary also [2]. There are reports suggesting a high risk in this group [6]. A carcinoma occurring in a fibroadenoma may be considered a chance occurrence of location as the epithelial component of a fibroadenoma is subjected to the same stimuli as rest of the breast parenchyma [3]. Azzopardi [4] suggested that carcinoma involving a fibroadenoma might be due to one of the following:

• Carcinoma arising in an adjacent breast tissue engulfing or infiltrating a fibroadenoma.

• Carcinoma in the crevices of a fibroadenoma as well as in the adjacent breast tissue

• Carcinoma restricted entirely, or at least dominantly, to a fibroadenoma as well as in the adjacent breast tissue.

In the presented case, however the malignancy had presented within the previously operated fibroadenoma that had recurred. In a comprehensive review of 26 cases published for the first time, it was observed that out of the reported 26 cases, 50% were *in situ *lobular carcinoma and 11% were infiltrating lobular carcinoma. In situ ductal carcinoma was reported in 22% of these cases [7].

Pick and Lossifides reviewed 62 cases of carcinoma in situ and in 42% cases surrounding breast carcinoma was involved as well [7]. It may be difficult to suspect the malignant transformation in a fibroadenoma as the clinical and radiological signs may be masked until breach of the false capsule and the diagnosis is invariably reached on histopathological examination of the tumor therefore high suspicion index is mandatory [8-11].

The mammographic features of carcinoma originating within a fibroadenoma include indistinct margins, clustered micro calcifications but it is quite difficult to differentiate the benign fibroadenomas from those harboring malignancy [12-15]

The surgical management would depend on the stage at presentation and the presence of axillary or distant metastasis. If the tumor is small (T1, T2) breast conservative surgery in the form of lumpectomy or wide local excision along with axillary lymph node dissection (in the N1 disease) may be optimum. Follow up including mammographic monitoring of both the breasts is recommended in view of the high incidence of contralateral breast cancer. There is also a recommendation of random biopsy of the contralateral breast in addition to treatment of the ipsilateral breast [8].

Prognosis depends on the grade and the stage at presentation but fibroadenoma may attract early attention leading to early detection and good outcome. Although the malignant transformation of a fibroadenoma is rare, the presence of this tumor in a woman with a positive family history may have greater clinical importance than fibroadenomas arising in women with no additional risk factors [11-15].

## Conclusion

Like in presented case one has to be suspicious of a fibroadenoma developing in a middle-aged woman with a family history of breast and/or ovarian cancer. One has to be aware of the progression capabilities of fibroadenomas, in particular in women with a BRCA mutation or a strong family history for breast/ovarian cancer. There is strong recommendation to histological evaluation of all breast masses in women with a strong positive family history for ovarian/breast cancer.

## Consent

Written informed consent was obtained from the patient for publication of this case report and accompanying images. A copy of the written consent is available for review by the journal's Editor-in-Chief.

## Competing interests

The authors declare that they have no competing interests.

## Authors' contributions

CM was the chief surgeon in charge of the case, PK and NS were the first and second assistants in the management of the case and the manuscript. DB, TA, MT, RK assisted in the preparation of manuscript. AB and SS were the pathologists responsible for the histopathology and immuno-histochemistry
